# High frequency of *β*-catenin heterozygous mutations in extra-abdominal fibromatosis: a potential molecular tool for disease management

**DOI:** 10.1038/sj.bjc.6605557

**Published:** 2010-03-02

**Authors:** J Dômont, S Salas, L Lacroix, V Brouste, P Saulnier, P Terrier, D Ranchère, A Neuville, A Leroux, L Guillou, R Sciot, F Collin, A Dufresne, J-Y Blay, A Le Cesne, J-M Coindre, S Bonvalot, J Bénard

**Affiliations:** 1 Sarcoma Committee, Institut Gustave Roussy, Villejuif, France; 2 Institut Bergonié, Bordeaux, France; 3 Translational Laboratory, Institut Gustave Roussy, Villejuif, France; 4 Department of Clinical Biology and Pathology, Institut Gustave Roussy, Villejuif, France; 5 Centre Léon Bérard, Lyon, France; 6 Department of Pathology, Hôpital Hautepierre, Strasbourg, France; 7 Department of Pathology, Centre Alexis Vautrin, Nancy, France; 8 University Institute of Pathology, Lausanne, Switzerland; 9 Department of Pathology, KU Leuven, Belgium; 10 Department of Pathology, Centre Georges-François Leclerc, Dijon, France; 11 INSERM U916, Pathology Department, University Victor Ségalen, Bordeaux, France; 12 CNRS-UMR 8126, IFR54, Institut Gustave Roussy, Villejuif, France

**Keywords:** *β*-catenin mutation, fibromatosis, prognostic factor

## Abstract

**Background:**

Fibromatosis comprises distinct clinical entities, including sporadic extra-abdominal fibromatosis, which have a high tendency for recurrence, even after adequate resection. There are no known molecular biomarkers of local recurrence. We searched for *β*-catenin mutations in a European multicentre series of fibromatosis tumours to relate *β*-catenin mutational status to disease outcome.

**Methods:**

Direct sequencing of exon 3 *β*-catenin gene was performed for 155 frozen fibromatosis tissues from all topographies. Correlation of outcome with mutation rate and type was performed on the extra-abdominal fibromatosis group (101 patients).

**Results:**

Mutations of *β*-catenin were detected in 83% of all cases. Among 101 extra-abdominal fibromatosis, similar mutation rates (87%) were observed, namely T41A (39.5%), S45P (9%), S45F (36.5%), and deletion (2%). None of the clinico-pathological parameters were found to be significantly associated with *β*-catenin mutational status. With a median follow-up of 62 months, 51 patients relapsed. Five-year recurrence-free survival was significantly worse in *β*-catenin-mutated tumours regardless of a specific genotype, compared with wild-type tumours (49 *vs* 75%, respectively, *P*=0.02).

**Conclusion:**

A high frequency (87%) of *β*-catenin mutation hallmarks extra-abdominal fibromatosis from a large multicentric retrospective study. Moreover, wild-type *β*-catenin seems to be an interesting prognostic marker that might be useful in the therapeutic management of extra-abdominal fibromatosis.

Fibromatoses are rare, locally invasive soft-tissue tumours composed of spindle cells that display a wide spectrum of aggressiveness. These star-shaped soft tissues, devoid of a capsule, invade surrounding structures and have a high propensity for local recurrence (Sobin and Wittekind, 2002).

Clinical forms of the disease depend on the tumour site: abdominal fibromatosis (deep or desmoid), which occurs on the abdominal wall of women in association with hormonal factors; intra-abdominal fibromatosis, some forms of which are associated with germ-like mutations of the adenomatous polyposis coli (APC) gene-associated Gardner syndrome; and extra-abdominal fibromatosis, which occurs in the head and neck, trunk, and limbs. Sporadic extra-abdominal fibromatosis has a high tendency for local recurrence, even after apparently adequate resection. In institutional retrospective studies, local failure rates at 5 years have ranged from 25 to 60% (Bonvalot *et al*, 2008). This wide range reflects the great variability of accrual, treatments, and follow-up in this rare disease, which has not yet been investigated in a randomised controlled study. Traditionally, patients undergo standard surgery, with the primary goal always being to achieve complete resection with negative margins, as indicated for sarcomas. However, Bonvalot *et al* (2008) recently questioned the need for systematically including surgery and other aggressive treatments in the first-line treatment of primary extra-abdominal desmoid tumours. In their study, tumour growth arrest occurred in two-thirds of patients managed non-surgically. A larger, multi-institutional study conducted recently also showed that a wait-and-see approach avoided the need for aggressive surgery and radiotherapy in the majority of patients with primary tumours (Fiore *et al*, 2009). Intrinsic biological characteristics of tumour cells and the host microenvironment, for example, could account for highly diverse outcomes.

To date, no predictive criteria (clinical, histological, or biological) that might help oncologists in the clinical management of patients with extra-abdominal fibromatosis have been identified. From a biological point of view, desmoids and extra-abdominal fibromatosis tumours typically harbour mutations of APC and/or *β*-catenin genes (Alman *et al*, 1997; Tejpar *et al*, 1999). Adenomatous polyposis coli is known to regulate the cytoplasmic level of *β*-catenin, which is involved in cell adhesion and has a key role in the Wnt (Wingless) signalling pathway. The phosphorylation of *β*-catenin on threonine 41 and serine 33, 37, and 45, encoded by exon 3 of the *β*-catenin gene, occurs when *β*-catenin binds to APC and leads to protein degradation through the ubiquitin–proteasome pathway (Orford *et al*, 1997; Rubinfeld *et al*, 1997). Adenomatous polyposis coli mutations give rise to *β*-catenin protein accumulation and induce Wnt pathway activation. *β*-Catenin gene missense mutations, in turn, lead to post-translational stabilisation of the protein and translocation from the cytoplasm to the nucleus, where *β*-catenin binds to the T-cell factor lymphoid enhancer family, resulting in transcription transactivation (Morin *et al*, 1997). Such mutations had only been described in small series of desmoid tumours until very recently when Lazar *et al* (2008) reported a high frequency of *β*-catenin mutations in the vast majority of fibromatoses.

In this study, we analysed a large series of extra-abdominal fibromatoses (with follow-up data spanning over 5 years) collected through the connective tissue cancer network *Conticanet* in France, Belgium, and Switzerland to evaluate whether *β*-catenin mutational status might constitute a valuable biomarker for recurrence that could improve the therapeutic management of such disabling tumours.

## Materials and methods

### Tumour material of patients

All tumour samples were collected through CONTICANET and stored in a centralised tumour data bank at the Bergonié Institute in Bordeaux, France, after obtaining approval from the appropriate ethics committees. This European fibromatosis tumour database contained exhaustive clinical information and follow-up data for over 5 years. From this tumour database, 187 eligible fibromatosis tumour tissues collected between December 1987 and January 2007 were tested in this retrospective study. Most of the patients with extra-abdominal fibromatosis had been surgically treated with curative intent. The tumour material comprised tumour samples from biopsies or microbiopsies performed using 16- or 18-gauge needles. The tissues had been obtained after surgery at diagnosis and snap-frozen in liquid nitrogen. Twenty-three formalin-fixed, paraffin-embedded (FFPE) tumour samples paired to frozen tumour samples were also obtained, as were 35-*μ*m-thick tumour slices flanked by 4-*μ*m sections from either frozen or FFPE fibromatosis tumours for use as histological controls (haematoxylin–eosin–saffron staining). The histological controls were analysed by pathologists specialised in soft-tissue tumours (Ph T and JMC). Over 90% of the histological features identified were typical of fibromatosis in all tumours analysed.

### *β*-Catenin gene sequencing

Following lysis of the 35 *μ*m tumour slices, nucleic acids were obtained using Qiagen column separation according to the manufacturer's instructions (Qiagen, Hilden, Germany). Available DNA for sequencing was obtained in 155 desmoid-type fibromatosis tumour biopsy specimens including all clinical forms of the disease. DNA lymphocytes from 96 anonymous individuals were used as non-mutated controls. The *CTNNB1* mutations located in exon 3 were analysed by direct DNA sequencing. The genomic DNA was first amplified by polymerase chain reaction (PCR) of over 35 cycles at an annealing temperature of 55°C (forward primers: 5′-ATTTGATGGAGTTGGACATGGC-3′ and reverse: 5′-CCAGCTACTTGTTCTTGAGTGAAGG-3′) as previously described (Delmas *et al*, 2007). All PCR amplimers were checked using 1% agarose gel electrophoresis. Sequencing reactions were performed using a BigDye terminator cycle sequencing kit and analysed on a 48-capillary 3730 DNA Analyzer (Applied Biosystems, Foster City, CA, USA). Exonic sequences were read and aligned using SeqScapeR software (Applied Biosystems). As reference for *β*-catenin gene, NM 00109822091 aligned on a sequence of chromosome 3 (NCBI 36) was used.

### Patient population and clinical data

The varying clinical forms of fibromatosis (sporadic tumours, intra-abdominal tumours associated with hereditary syndrome, and deep and extra-abdominal tumours) were all represented in the *Conticanet* series of tumours used for this study. Of 155 fibromatosis tissues from all topologies available for DNA sequencing, 101 extra-abdominal fibromatoses were used to investigate the correlation between *β*-catenin mutational status and clinical characteristics: age, sex, tumour size, tumour location (head and neck, abdominal and thoracic walls and limbs), therapy (including quality of surgical resection as assessed by type of resection (R0, R1, or R2 according to the UICC-R classification system)) (Lev *et al*, 2007), and outcome (for 101 patients treated with a curative intent). R0 resection was obtained for 42 patients.

### Statistical analysis

Survival times were calculated from the date of diagnosis to the date of relapse (disease-free survival). Log-rank tests were used for survival outcome analysis. Descriptive statistics were used to correlate patient characteristics to the *β*-catenin genotype, and the relationship between clinical characteristics and *β*-catenin mutational status was analysed using logistic regression analysis.

## Results

### High frequency of *β*-catenin gene mutation in frozen tumour specimens

Of the 155 sequenced DNA from tumour samples obtained from the Conticanet fibromatosis tumour database, 129 (83%) contained a mutation of *β*-catenin exon 3. No mutations were detected in the DNA extracted from lymphocytes of the 96 controls. All sequences were controlled in an independent experiment. The rate of mutation identified (83%) confirms and updates the findings of a preliminary report by our group on the basis of 95 cases from the same series (Domont *et al*, 2009).

**Figure 1:**
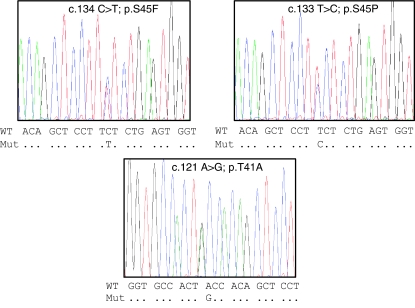
*β*-Catenin mutations detected in extra-abdominal fibromatosis tumours. Typical heterozygous missense mutations found in exon 3 *β*-catenin gene at codons 41 (T41A) and 45 (S45F and S45P).

*β*-Catenin missense mutations were essentially found at codons 41 and 45, identified as c.121 A>G or pThr-41-Ala (T41A), c.133T>C or pSer45Pro (S45P), and c.134C>T or pSer45Phe (S45F). The 41A and S45F mutations were relatively common, occurring in 41% (64 out of 155) and 34% (53 out of 155) of cases, whereas the S45P mutation occurred in just 6% (9 out of 155) (Figure 1). In three cases (2%), we also detected a small in-frame deletion of 3, 6, and 30 base pairs, which removed codon 36, codons 33 and 34, and codons 45–54, respectively.

### Potential diagnostic interest of mutation analysis on formalin-fixed tissues

The clinical potential of the above results obtained from frozen tumour tissues led us to test the feasibility of sequencing using FFPE tissues, which are more readily available in daily practice. We therefore compared sequencing results from the FFPE samples paired to the frozen tumour specimens of the series. We observed identical data in 20 out of 23 (87%) samples, with no false positives and just three false negatives from FFPE tissues. Archival FFPE tumour tissues might thus provide useful and easily obtainable material for determining *β*-catenin mutational status in daily practice.

### Characteristics of patients with extra-abdominal fibromatosis by *β*-catenin genotype

The European fibromatosis tumour database contained exhaustive clinical information, with follow-up data for over 5 years, for 101 patients (36 men) with extra-abdominal fibromatosis that had been surgically treated with curative intent. Fifty-seven patients had been treated for primary fibromatosis (57%) and 40 for relapse (40%) (Table 1). The *β*-catenin mutation rate detected in this group (87% (88 out of 101)) was similar to that detected for the whole series, as was the distribution of mutation types (T41A, 39.5% (40 out of 101), S45F, 36.5% (37 out of 101), and S45P, 9% (9 out of 101)) (Figure 1). We also found two deletions (2%). Table 1 shows patient characteristics according to *β*-catenin mutational status. Most of the surgical margins were R0 (42%) or R1 (34%). Few patients had received postoperative radiation therapy (18%) or medical treatment (8%). With a median follow-up of 62 months (range 3–452), 51 patients (51%) relapsed. *β*-Catenin mutational status was not found to be significantly associated with sex, age, tumour site, presentation (primary or relapse), or therapy. No correlation could be established between surgical resection quality and mutation rate.

### Correlation of outcome with mutation rate and type

**Figure 2:**
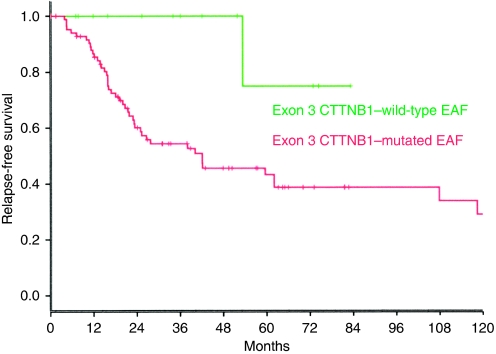
Kaplan–Meier graph of relapse-free rate–time as a function of a mutation event. Plots of time from time of first surgery to remove tumour (primary or relapse) of extra-abdominal fibromatosis (EAF) to the first recurrence event according to Kaplan–Meier for the entire cohort (*n*=101). Plot of time from tumour surgery (primary or relapse) to recurrence event. Analysis reveals that the mutated genotype, irrespective of the *β*-catenin mutation (T41A, S45F, or S45P), is a significant predictor of relapse (*P*=0.02).

**Figure 3:**
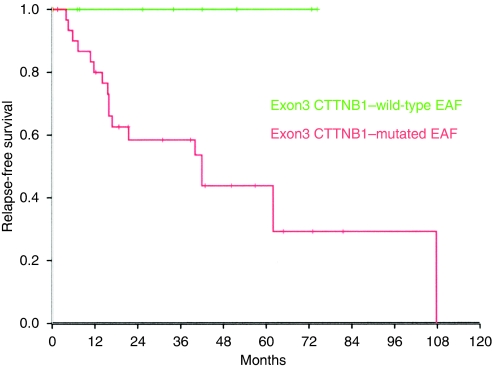
Kaplan–Meier representation of relapse-free rate–time as a function of a mutation event for patients operated for extra-abdominal fibromatosis (EAF) with RO resection (*n*=42). Plot of time from tumour surgery (primary or relapse) to recurrence event. Multivariate analysis reveals that the *β*-catenin-mutated genotype, irrespective of the mutation (T41A, S45F, or S45P), is a significant predictor of relapse (*P*=0.02).

To test whether *β*-catenin mutational status correlated with clinical outcome in patients with extra-abdominal fibromatosis, we compared the frequency of disease-free survival (estimated from the date of diagnosis to the date of relapse) in patients with wild-type and *β*-catenin-mutated tumours. Among 51 relapses (51% of the cohort), 1 and 50 were from patients with wild-type tumour and mutated tumour, respectively. Five-year recurrence-free survival (Figure 2) was significantly shorter in all *β*-catenin-mutated tumours (43%), compared with wild-type tumours (75%) (*P*=0.02). We then attempted to relate poor outcome to a specific genotype but found no significant differences for T41A, S45F, or S45P mutants, although the 45F mutation did seem to show a weak trend of significance for poorer outcome. Given the possible prognostic impact of surgical margin quality, we analysed the *β*-catenin genotype in patients with R0 resection (*n*=42) and found relapse to be significantly higher in patients with mutated tumours (18 out of 33) than wild-type tumours (0 out of 9) (*P*=0.02), as shown in the Kaplan–Meier graph (Figure 3).

## Discussion

As dysregulation of expression and mutation of *β*-catenin hallmarks sporadic aggressive fibromatosis (Tejpar *et al*, 1999), we searched for exon 3 *β*-catenin gene mutations in a large European multicentre series of extra-abdominal fibromatosis tumours. Indeed, mutations of exon 3 of *β*-catenin, which harbours phosphorylable serine/threonine residues, induce stabilisation of *β*-catenin and subsequent biological properties such as proliferation and invasiveness.

Our study revealed a high frequency of *β*-catenin mutations in fibromatosis tumours (*n*=155), and in extra-abdominal forms in particular (*n*=101), results similar to that reported by another study (Lazar *et al*, 2008) that included a similar-sized series of fibromatosis tumours (*n*=138). Remarkably, all mutations clustered at Threonine 41 (T41A) and Serine 45 (S45F and S45P) in all topographies, suggesting that these residues have crucial functions in the *β*-catenin/Wnt signalling pathway required for the maintenance of fibroblast/myofibroblast homoeostasis (Cheon *et al*, 2002; Clevers, 2006; Polakis, 2007). Irrespective of the molecular mechanism at work behind these two specific residues (Xu *et al*, 2007), a high frequency of mutations (87%) hallmarks extra-abdominal fibromatosis at a molecular level. On studying a series of 101 such tumours, we found that 5-year recurrence-free survival was significantly worse in all mutated tumours (43%) compared with wild-type tumours (75%) (*P*=0.02). However, no specific mutant genotypes (T41A, S45F, or S45P mutants) were related to prognosis, although we did observe a weak trend for S45F, but it was far from significant. Our results contrast with those of a very recent American study proposing that patients with fibromatosis harbouring an S45F mutation are at particular risk of recurrence (Lazar *et al*, 2008). The discrepancy may reflect differences in the clinical entities studied. Although our study was restricted to extra-abdominal tumours, Lazar *et al* (2008) also included deep abdominal tumours.

Evidence that *β*-catenin mutations influence the risk of relapse is further strengthened by our analysis of patients with R0 resections, in which relapse-free survival was significantly higher in wild-type than in mutated tumours. Hence, as extra-abdominal fibromatosis relapse is clinically unpredictable, its risk of occurrence could be evaluated using *β*-catenin mutational status: a wild-type tumour, for example, would predict good outcome, whereas a *β*-catenin mutation would predict a potential risk of relapse. It is probably not the only factor involved, however, as wild-type tumours represent 16% of cases in our study, whereas indolent extra-abdominal fibromatosis represents roughly 50% of the patients according to a recent multi-institutional European analysis (Fiore *et al*, 2009). Therefore, several mutated tumours harbouring stabilised *β*-catenin do not relapse. Various mechanisms could explain this paradox. First of all, it is possible that deregulation of additional molecular actors of the canonical Wnt pathway – from the receptor (with agonists and antagonists) to cytoplasmic effectors and finally to the target gene – may contribute to the tumour phenotype. Second, alteration of the non-canonical Wnt pathway and effectors must also be considered. Hence, additional prospective studies are needed to find more discriminant markers.

Finally, from a practical point of view, the feasibility test comparing sequences from frozen and FFPE tumour tissues showed that *β*-catenin mutational status can be assessed using either fresh or archival extra-abdominal tumour specimens in accordance with the tumour collection system used in each pathology laboratory. This simple yet efficient molecular test might prove to be a very interesting complementary diagnostic tool to regular histological examination in extra-abdominal fibromatosis. Indeed, in cases in which there is insufficient tumour material for histological analysis, *β*-catenin sequencing could be performed, as it requires just a few ng of gDNA. The test could also be helpful to resolve puzzling cases in which the histological interpretation of primary tumour tissue obtained by fine-needle aspiration is complicated.

A final application would be to use molecular tumour findings to individualise the choice of management protocols. This application of personalised medicine in patients with extra-abdominal fibromatosis could help to identify the right treatment at the right time for optimal outcome.

## Figures and Tables

**Table 1:** Characteristics of patients with extra-abdominal fibromatosis by *β*-catenin genotype

***CTNNB1***	**Total**	**WT**	**Mutated**	**41A**	**45F**	**45P**	**del**
**characteristics**		***n***	***n***	***n***	***n***	***n***	***n***
*N*	101	13	88	40	37	9	2
							
*Age*							
Median, years	37	39.5	37.5	36.7	37	45.5	43
Range	0.1–77	0.5–66	0.1–77	10–73	0.1–77	14–65	19–68
							
*Sex*							
Male	36	4	32	14	15	3	—
Female	65	9	56	26	22	6	2
							
*Presentation*							
Primary	57	12	45	16	20	8	1
Relapse	40	1	39	22	15	**1**	**1**
NA	4	—	4	2	2	—	—
							
*Tumour site*							
Head/Neck	8	1	7	2	5	—	—
Trunk	54	5	49	20	19	8	2
Limb	37	7	30	17	12	1	—
NA	2	—	2	1	1	—	—
							
*Tumour size, mm*							
Median, mm	80	40	80	80	85	75	40
Range	10–300	15–120	10–300	21–300	25–200	25–190	10–70
							
*Therapy*							
Surgery	101	13	88	40	37	9	2
Radiation therapy	18	1	17	7	8	1	1
Medical therapy	8	1	7	3	4	—	—
							
*Surgical margin*							
RO	42	9	33	14	13	5	1
R1	33	3	30	15	12	3	—
R2	8	—	8	1	5	1	1
NA	18	1	17	10	7	—	—
							
*Outcome*							
Relapse	51	1	50	22	23	4	1

Patient clinical data and *β*-catenin genotype tumour data from the series of extra-abdominal fibromatosis tumours. WT, wild *β*-catenin gene (exon 3) del, deletion in exon 3; 41A, 45F, 45P, mutated residues; n, number of patients; NA, not available; mm, millimiter; R0, R1, and R2, quality of surgical margins according to the UICC-R classification system.
